# Supplementary material for: Modulation of Structural and Physical-Chemical Properties of Fish Gelatin Hydrogel by Natural Polysaccharides
Source: Int J Mol Sci. 2025 Mar 22;26(7):2901. doi: 10.3390/ijms26072901 (PMC11988395; doi:10.3390/ijms26072901)
Supplement: Supplementary file 1 [file ijms-26-02901-s001.zip › ijms-3520270-supplementary.pdf]

Supporting Information

Modulation of structural and physical-chemical properties of fish gelatin hydrogel by natural polysaccharides

Aidar T. Gubaidullin [1,2,*], Aliya I. Galeeva [3], Yuriy G. Galyametdinov [3], Georgiy G. Ageev [4], Alexey A. Piryazev [4], Dimitri A. Ivanov [4,5], Elena A. Ermakova [1], Alena A. Nikiforova [1,6], Svetlana R. Derkach [7], Olga S. Zueva [8] and Yuriy F. Zuev [1,*]

[1] Kazan Institute of Biochemistry and Biophysics, FRC Kazan Scientific Center of RAS, Lobachevsky St., 2/31, 420111 Kazan, Russia; yufzuev@mail.ru (Y.F.Z.)
[2] Arbuzov Institute of Organic and Physical Chemistry, FRC Kazan Scientific Center of RAS, Arbuzov Street 8, 420088 Kazan, Russia; aidar@iopc.ru (A.T.G.)
[3] Physical and Colloid Chemistry Department, Kazan National Research Technological University, 420015 Kazan, Russia; galeeva-alija@mail.ru (A.I.G.); yugal2002@mail.ru (Y.G.G.)
[4] Scientific Center for Genetics and Life Sciences Sirius University of Science and Technology, Olympic Avenue, 1, 354340 Sochi, Russia; ageev.gg@talantiuspeh.ru (G.G.A.); piryazev.aa@talantiuspeh.ru (A.A.P.); ivanov.da@talantiuspeh.ru (D.A.I.)
[5] Institut de Sciences des Matériaux de Mulhouse–IS2M, CNRS UMR 7361, F-68057 Mulhouse, France (D.A.I.)
[6] A. Butlerov Chemical Institute, Kazan Federal University, Kremlevskaya St. 18, 420008 Kazan, Russia; alnikiforova22@gmail.com (A.A.N.)
[7] Institute of Natural Sciences and Technology, Murmansk Arctic University, Sportivnaya Str. 13, Murmansk 183010, Russia; derkachsr@mauniver.ru (S.R.D.)
[8] Institute of Electric Power Engineering and Electronics, Kazan State Power Engineering University, Krasnoselskaya St. 51, 420066 Kazan, Russia; ostefzueva@mail.ru (O.S.Z.)
* Correspondence: aidar@iopc.ru (A.T.G.); yufzuev@mail.ru (Y.F.Z.)

Contents

General Remarks……………………………………………………………………………………………S2

Figure S1. Experimental PXRD curves for fish gelatin (a) and combined systems of k-carrageenan-gelatin (b) at temperatures 1°C (blue), 26°C (green) and 45°C (red)…………………………………………………...S2

Figure S2. Experimental PXRD curves of for combined systems of alginate-gelatin (a) and combined systems of chitosan-gelatin (b) at temperatures 1°C(blue), 26°C (green) and 45°C (red)……………..……S2

Figure S3. Experimental PXRD curves of fish gelatin and combined systems with k-carrageenan, alginate and chitosan at temperatures (a) - 1°C(blue), (b) - 26°C (green) and (c) - 45°C (red)……………….………S3

Figure S4. Fitting of experimental curves in monodisperse spherical approximation (logarithmic scale) for fish gelatin at temperatures 1°C (a), 26°C (b) and 45°C (c). Experimental data are shown in dots, lines are fitting curves. Scattering vector $s = 4\pi \mathrm{Sin}\theta/\lambda$, $Å^{-1}$; $\lambda = 1.5418Å$ is the X-ray wavelength……………………………………………………………………………………………….S3

Figure S5. Fitting of experimental data in monodisperse spherical approximation for combined systems with k-carrageenan (a), alginate (b) and chitosan (c) at temperatures 1°C for determination of the particle distance distribution functions p(r)). Scattering vector $s = 4\pi \mathrm{Sin}\theta/\lambda$, $Å^{-1}$; $\lambda = 1.5418Å$ is the X-ray wavelength)……………………………………………………………………………………………S3

References ………………………………………………………………………………………….….S3

General Remarks

Small-angle X-ray scattering (SAXS) and partly Wide-angle X-ray scattering (WAXS) were performed in the Sirius University of Science and Technology, Research Center for Genetics and Life Sciences (Sochi, Russian Federation) using a combined SAXS/WAXS XeuSS diffractometer (Xenocs, France), (Genix3D source, Pilatus 300k two-dimensional detector, CuKα radiation, λ=1.5418Å). Experiments were carried out at temperatures of 1°, 26° and 45°C. Samples in liquid state were placed in the borosilicate glass capillaries with 1.5 mm or 2 mm diameter, with low X-ray absorption coefficient. The sample-to-detector distance was 5 cm in WAXS and 70 cm in SAXS modes. These distances were calibrated using silver behenate as a calibration standard.

WAXS experiments of gel samples in real time (during their drying on the surface of silicon wafer) were made in Distributed Spectral-Analytical Center of Shared Facilities for Study of Structure, Composition and Properties of Substances and Materials of FRC Kazan Scientific Center of RAS using the Bruker D8 Advance diffractometer, equipped with the Vario attachment and Vantec linear PSD using Cu K$_{\alpha1}$ radiation (40 kV, 40 mA), monochromated by a curved Johansson monochromator (λ = 1.5406 Å). Data were collected in reflection modes, liquid samples were placed on the surface of standard silicon plate with zero diffraction, which reduces the background scattering. The samples were kept spinning (15 rpm) throughout the data collection. Patterns were recorded in the 2θ range between 3 and 90° with 0.008° steps and step time of 0.1–4.0 s. Several diffraction patterns in various experimental modes were collected for the samples. Data processing was performed using the EVA and TOPAS software packages [1,2].

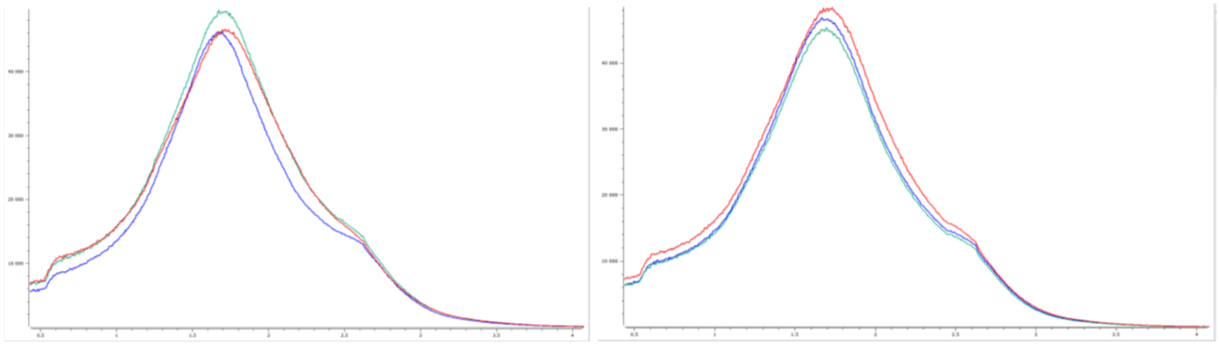

a **b**

Figure S1. Experimental PXRD curves for fish gelatin (a) and combined systems of k-carrageenan-gelatin (b) at temperatures 1°C (blue), 26°C (green) and 45°C (red)

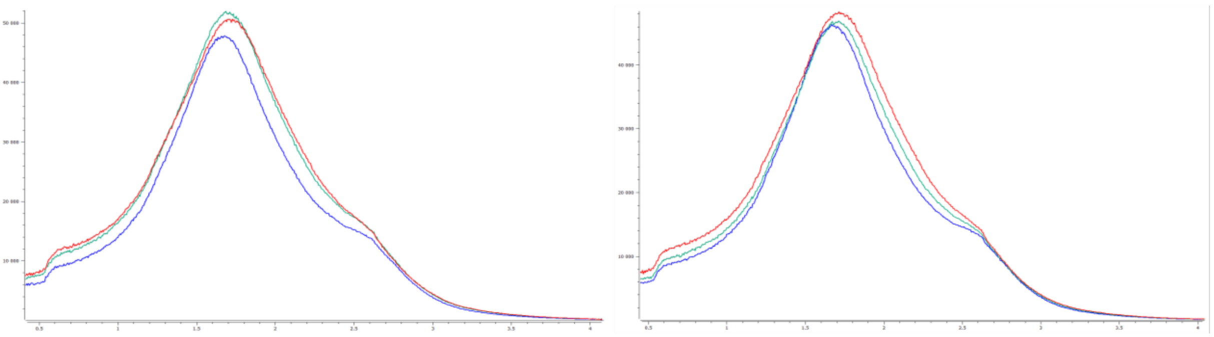

a **b**

Figure S2. Experimental PXRD curves of for combined systems of alginate-gelatin (a) and combined systems of chitosan-gelatin (b) at temperatures 1°C(blue), 26°C (green) and 45°C (red).

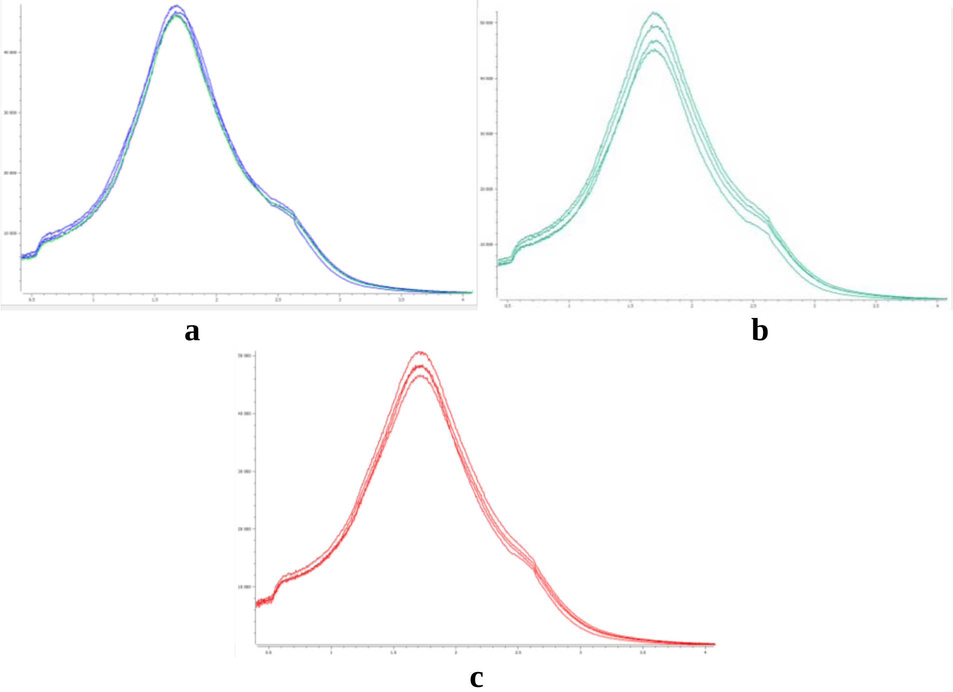

Figure S3. Experimental PXRD curves of fish gelatin and combined systems with k-carrageenan, alginate and chitosan at temperatures (a) - 1°C(blue), (b) - 26°C (green) and (c) - 45°C (red).

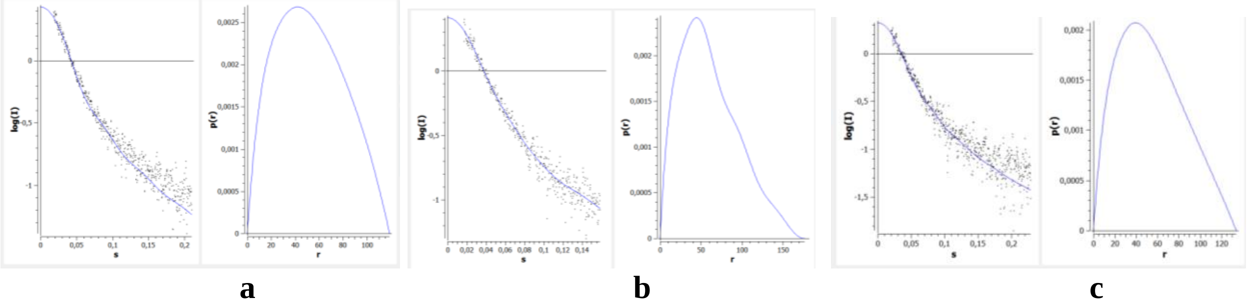

Figure S4. Fitting of experimental curves in monodisperse spherical approximation (logarithmic scale) for fish gelatin at temperatures 1°C (a), 26°C (b) and 45°C (c). Experimental data are shown in dots, lines are fitting curves. Scattering vector $s = 4\pi \mathrm{Sin}\theta/\lambda$, Å^{-1}; λ = 1.5418Å is the X-ray wavelength.

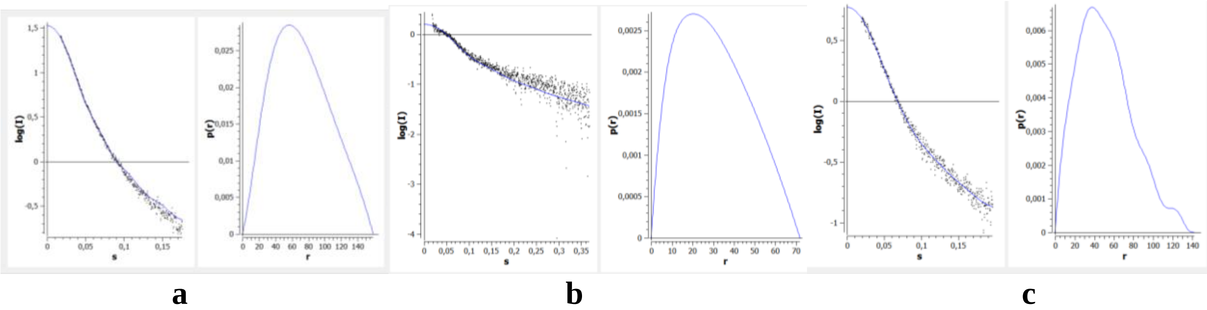

Figure S5. Fitting of experimental data in monodisperse spherical approximation for combined systems with k-carrageenan (a), alginate (b) and chitosan (c) at temperatures 1°C for determination of the particle distance distribution functions p(r)). Scattering vector $s = 4\pi \mathrm{Sin}\theta/\lambda$, Å^{-1}; λ = 1.5418Å is the X-ray wavelength.

References

(1) DIFFRAC Plus Evaluation package EVA, Version 11 (2005). User's Manual, Bruker AXS, Karlsruhe. Germany. - 258 p.

(2) *TOPAS V3: General Profile and Structure Analysis Software for Powder Diffraction Data, Technical Reference,* Bruker AXS: Karlsruhe, Germany, 2005.
